# Health Behavior and Social-Emotional Health Status of School-Aged Children According to their Experience with Atopic Dermatitis Diagnosis: Based on the 12th (2019) Panel Study on Korean Children

**DOI:** 10.3390/children10020288

**Published:** 2023-02-02

**Authors:** Da-Jeong Kum, Kyung-Sook Bang

**Affiliations:** 1 College of Nursing, Seoul National University, Seoul 03080, Republic of Korea; 2 College of Nursing, The Research Institute of Nursing Science, Seoul National University, Seoul 03080, Republic of Korea

**Keywords:** atopic dermatitis, health behavior, social-emotional health status, school-aged children, PSKC

## Abstract

Atopic dermatitis (AD) is a chronic inflammatory skin disease that is common in children and is increasing worldwide. This study aimed to identify differences in children’s health behavior and social-emotional health status based on AD diagnosis at late school age. For this purpose, we conducted a descriptive survey using the 12th Panel Study on Korean Children data obtained in 2019. The data were analyzed using descriptive statistics, the Rao–Scott $\chi^{2}$ test, and a t-test using a complex sample analysis. A total of 1412 11-year-old Korean children participated in the study, of whom an estimated 8.2% were diagnosed with AD. In the children diagnosed with AD, the transition from exclusive breastfeeding to mixed feeding was later than that in children without AD (F = 5.71, *p* = 0.024), and the prevalence of AD in their parents was higher (F = 6.97, *p* = 0.014). Regarding health behaviors, the children diagnosed with AD had a higher intake frequency of protein (F = 5.41, *p* = 0.028) and vegetables (F = 6.09, *p* = 0.020). Regarding social-emotional health, subjective health status (F = 3.94, *p* = 0.026) and friend relationships (F = 2.95, *p* = 0.007) were lower in the children diagnosed with AD. These results, as preliminary data for interventions for school-aged children with AD, suggest that the difficulties of children’s peer relationships should be considered and dealt with in further interventions.

## 1. Introduction

Atopic dermatitis (AD) is a chronic inflammatory skin disease that is characterized by itching and eczema. It is a common allergic disease in children, along with asthma and allergic rhinitis. In Korea, the prevalence of AD in children is 13.5%, and it is steadily increasing [1,2]. Children with AD have a poor quality of life due to skin damage and itching, and their parents also suffer from fatigue and depression due to their high care needs [3]. Therefore, active interventions are required to relieve AD symptoms and to improve the quality of life of patients.

The cause of AD has not been clearly identified, but it has been reported to be affected by diet, environmental factors, and lifestyle choices. AD is difficult to treat, and symptoms persist through repeated deterioration and alleviation, influenced by factors such as living environment and stress. Therefore, to manage AD, it is important to maintain a healthy lifestyle, to keep the skin clean and dry by using appropriate bathing techniques and moisturizers, and to remove irritation that causes itching [4]. Furthermore, topical steroid and immunosuppressive ointments have proven to be effective treatments [4].

AD requires children to manage their daily lives in order to control allergens and relieve symptoms, which affect health behaviors, such as eating and exercise. Food allergies are recognized as the main causes of the worsening of AD, and approximately 60% of children with AD have food restrictions [5]. Increasing physical activity is an important component of a healthy lifestyle, and it has been shown to reduce stress in adolescents with AD [6]. However, because physical activity aggravates AD symptoms, children suffering from AD tend to have a lower exercise capacity [7]. In addition, AD has been reported to be aggravated in cases of overweight or obesity and in cases of excessive sedentary time [8]. Moreover, children with AD sleep insufficiently due to itching, and this leads to the worsening of symptoms [8].

As a result of AD, various behaviors change, which can affect not only the disease itself but also overall health; therefore, it should be carefully examined. Children of late school age begin to understand their health conditions related to their chronic diseases and acquire various skills to manage them, and the health behaviors learned during this period are easily integrated into their lives and persist into adulthood [9]. Therefore, it is important to see that children with AD are able to maintain proper health behaviors and manage their AD.

Chronic diseases also have an impact on children’s daily and school lives, as well as on their self-concepts and peer relationships [10]. In particular, externally exposed AD lesions have a negative impact on a child’s body image, causing social alienation and difficulties in school life [3,11]. Late school age is a critical period in the development of a child’s self-concept. Therefore, children’s social-emotional health must be assessed, and active interventions are required to improve school adaptation and to build positive self-concepts.

In this study, we examined the differences between children’s health behavior and social-emotional health status based on AD diagnosis during late school age in order to develop an active intervention. We used data from the Panel Study of Korean Children (PSKC), an annual panel study of newborns sampled throughout Korea that began in 2008 and will run until 2027 [12]. The PSKC deals with community and policy characteristics, as well as children’s daily life characteristics, physical and health characteristics, and cognitive and emotional development. Since the participants entered school age from the 8th survey (2015), this study used data from the latest 12th survey (2019) to study children of late school age with and without AD.

## 2. Materials and Methods

### 2.1. Research Design

This descriptive survey study used secondary data from the 12th (2019) PSKC to identify the effect of AD diagnosis on the health behaviors and social-emotional health of school-going children in Korea.

### 2.2. Data Source and Study Population

The PSCK is a nationwide survey conducted annually to collect and provide longitudinal data on the growth and developmental characteristics of children, parenting status and needs, the functions and effects of childcare support institutions, and the effects of local communities and childcare policies at the national level [12]. The survey population consists of newborns born between April and July 2008 in targeted hospitals nationwide, and the criterion for the target hospitals was that it should have more than 500 deliveries in 2006. Sample extraction was performed via stratified multistage extraction to represent the Korean population, and the total sample comprised 2150 households. In the panel extraction process, cases were excluded when the mother was under the age of 18, the mother or the newborn was seriously ill, the child was part of a multiple birth, the mother had difficulty in communicating, or the child was scheduled to be adopted.

The sample of this study included children aged 11 years (5th grade in elementary school) who participated in the 12th (2019) PSKC survey. A total of 1417 households participated in the 12th (2019) PSKC survey, and 1412 households that responded to the diagnosis of AD were included in the final analysis. The variables of the study were based on the 12th (2019) survey, but among the general characteristics, the infant feeding type and the start of mixed feeding information were collected from the 7th (2014) survey, and the parents’ AD diagnosis information was collected from the 8th (2015) survey.

The PSKC was conducted with the approval of the Institutional Review Board of the Korea Institute of Child Care and Education (KICCE) (IRB No. KICCEIRB-2019-NO.01), and this study was approved by the Institutional Review Committee of the researchers’ affiliated university (IRB No. E2212/003-001). This study was conducted by downloading raw data from the KICCE website. The data were anonymized and available to the public.

### 2.3. Study Variables

#### 2.3.1. Characteristics of Children and Parents

The general characteristics of the children were sex, height, weight, body mass index (BMI; kg/m^2^), initial lactation type, and AD diagnosis. Weight and height were measured by the PSKC investigator, and BMI was calculated using the measured weight and height. According to the 2017 Korean National Growth Charts for children and adolescents, those with a BMI below the 5th percentile for a given age are classified as underweight, those with a BMI above the 5th and below the 85th percentile for a given age are classified as normal, those with a BMI above the 85th percentile and below the 95th percentile for a given age are classified as overweight, and those with a BMI above the 95th percentile for a given age are classified as obese [13]. AD diagnosis was classified as “yes” if the parents reported the date when their child was first diagnosed with AD, and “no” otherwise. The initial lactation types were infant feeding and the start of mixed feeding. The infant feeding type was classified into exclusive breastfeeding, mixed feeding, and formula feeding based on the method used up to the first six months reported in the 7th survey by their mother, and the start of mixed feeding was classified as “0–2 months,” “2–4 months,” and “5–6 months” based on the 7th survey as reported by the mothers. The general characteristics of the parents included their employment status and AD diagnosis result obtained from the 12th and 7th survey data, respectively. The employment status of the parents was classified as “employment,” “student,” and “neither”.

#### 2.3.2. Health Behaviors of Children

Health behaviors included children’s dietary patterns, physical activity, and sleep hours. First, dietary pattern was evaluated based on the frequency of intake per food group and the frequency of intake of carbonated drinks and instant food. In order to examine the frequency of intake by food group, the mothers answered the questions related to their child’s eating habits with “Agree,” “Neutral,” and “Disagree” for each food group. The intake of four recommended types of food (dairy products, protein, vegetables, and fruits) and the intake of four restricted items (oil, fat, salt, and snacks) were examined by asking ten questions: “drinking more than two bottles of dairy products every day,” “eats protein including meat, fish, egg, bean, tofu, and etc. every meal,” “eat vegetables other than kimchi in every meal,” “eat one fruit or a cup of fruit juice every day,” “eat fried dishes more than twice a week,” “eat fatty meat and fish at least twice a week,” “add more salt or soy sauce to the food when you eat,” and “eat ice cream, cake, snacks, and soda more than twice a week as a snack.” The frequencies of soda and instant food intake were identified by the children’s own responses: “not drinking in the last seven days,” “one to two times a week,” “two to three times a week,” and “more than five times a week”.

Physical activity was measured by the number of days of practice for more than 30 min and sedentary time during the day. Days of physical activity for more than 30 min were reported by the mothers as ranging between “none (1 point),” “1 day (2 points),” “2 days (3 points),” “3 days (4 points),” “4 days (5 points),” and “5 days or more (6 points)” for both indoor and outdoor physical activities. Sedentary time was the sum of time spent studying alone, taking online education courses, reading books, watching videos, and playing games, as reported by the mothers.

Sleep time included the child’s weekday and weekend sleep hours, which were calculated based on the child’s wake-up time and bedtime on weekdays and weekends reported by their mothers.

#### 2.3.3. Social-Emotional Health of Children

Social-emotional health status included resilience, overall happiness, body esteem, subjective health status, relationships with friends, relationships with teachers, bullying experience, and academic performance.

Resilience was measured using the Ego Resilience Scale developed by Block and Kremen [14] and translated by Yoo and Shim [15]. The tool has 13 questions, and the children responded on a 4-point Likert scale for each question, ranging from “not at all (1 point)” to “very much (4 points),” with higher scores indicating higher self-elasticity. In a previous study [15], the tool’s Cronbach’s α was 0.67, and it was 0.85 in this study. 

Overall happiness was measured by translating and modifying the children’s happiness questionnaire from The Millennium Cohort Study [16]. The questionnaire evaluates children’s happiness with school study, appearance, family, friends, school, and life with six questions. The original tool uses a 7-point Likert scale, but it was revised to a 4-point Likert scale in the PSKC, and an additional facial expression picture was presented for a better understanding of the children. Children responded to the 4-point Likert scale from “I’m not happy at all (1 point)” to “I’m very happy (4 points),” with a higher score indicating a high overall happiness. The questionnaire’s Cronbach’s α was 0.84 in a previous study [17], and it was 0.76 in this study.

Body esteem was measured using the revised Body-Esteem Scale by Mendelson and White [18]. Four of the twenty-four questions of the scale related to appearance and body satisfaction were used in this study, and five additional questions were added after expert review. Children responded on a 4-point Likert scale from “not at all (1 point)” to “very much (4 points),” with a higher score indicating positive body esteem. In the study conducted by Mendelson and White [18], the split-half reliability (r) of the tool divided by odd and even numbers was 0.85 (*p* < 0.002), and Cronbach’s α in this study was 0.70.

Subjective health status was answered by the mothers on a 5-point Likert scale from “very unhealthy (1 point)” to “very healthy (5 points)”.

Relationships with friends and teachers were identified by the school adaptation scale invented by Min [19] and revised by the PSKC. In the PSKC, five questions related to school events were eliminated, and some of the teacher-related questions were modified. The school adaptation scale consists of four sub-factors: learning activity, school rules, friendship, and teacher relationships. There are a total of 21 questions. This study used six friendship questions and five teacher-relationship questions. Children responded on a 4-point Likert scale from ”not at all (1 point)” to ”very much (4 points),” with a higher score indicating a better relationship. In a previous study [20], Cronbach’s α was 0.68 for friendship and 0.89 for teacher relationships, and in this study, each value was the same, at 0.68 and 0.89, respectively.

Bullying experience was measured by children’s responses to seven bullying situations, and each was answered by “never experienced (0 points),” “1 to 2 times a year (1 point),” “1 to 2 times a month (2 points),” “2 to 3 times a month (3 points),” “1 to 2 times a week (4 points),” and “multiple times a week (5 points)”.

Teachers assessed academic performance using questions modified to suit the academic level of the panel children based on the tool for comprehensive childcare service evaluation [21]. Teachers responded to 15 questions about Korean, English, mathematics, science, society, arts, and sports on a 5-point Likert scale from “bottom 20% (1 point)” to “top 20% (5 points),” where a higher score indicates higher academic achievement of the children. Cronbach’s α was 0.96 for a previous study [17], as well as for the current study.

#### 2.3.4. Data Analysis

The PSKC data are stratified cluster samples and should be analyzed using a complex sample analysis by applying stratification variables, clustering variables, and weights [12]. We applied a complex sample analysis using weights to make the results more representative and accurate. Stratification and clustering variables were identified according to the sampling methods, and the weights were calculated for each year’s PSKC data. The cross-sectional weight presented was used when analyzing the 12th data of the PSKC only, and the integrated weight was calculated and used when analyzing two different years’ data (7th and 12th data for feeding information, 8th and 12th data for parents’ AD diagnosis information). All data were analyzed using SPSS version 25.0 (IBM Corp., New York, NY, USA).

Descriptive statistics are presented as unweighted frequency, weighted percentage, estimated mean, and standard error. Differences in the general characteristics of the children and parents, health behaviors, and the social-emotional health of the children diagnosed with AD were analyzed using the Rao–Scott $\chi^{2}$ test and t-test.

## 3. Results

### 3.1. Comparison of General Characteristics in Atopic Dermatitis Group and Non-Atopic Dermatitis Group

A total of 1412 children who responded to the diagnosis of AD selected as the final participants of this study, representing the population of 469,015 11-year-old Korean children. Among the participants, 8.2% had a history of AD, and 91.8% had no history of AD (non-AD); the rates of normal BMI were the highest in the children at 78.4% and 69.7%, respectively, followed by overweight or obese (16.6% in the AD group, and 24.1% in the non-AD group), but the difference was not statistically significant. In the AD and non-AD groups, the rates of exclusive breastfeeding were the highest at 49.5% and 47.5%, respectively, and there was no difference between the two groups. Regarding the change from exclusive breastfeeding to mixed feeding, the start of mixed feeding was significantly late in the AD group, with a rate of 35.9%, which was 17.5% in the non-AD group (F = 5.71, *p* = 0.024). The rates of parents diagnosed with AD were 19.6% and 9.2% in the AD and non-AD groups, respectively (F = 6.97; *p* = 0.014) (Table 1).

### 3.2. Differences in Health Behaviors by History of Atopic Dermatitis Diagnosis

The intake frequency of the recommended food was generally high in the AD group, especially that of protein (F = 5.41, *p* = 0.028) and vegetables (F = 6.09, *p* = 0.020). The intake frequency of restricted food was also somewhat higher in the AD group; however, the difference was not statistically significant. There was no statistically significant difference in soda and instant food intake; however, the rates of not consuming soda and instant food were somewhat higher in the non-AD group. There were no statistically significant differences in the frequency of physical activity, static activity time, and sleep time between the two groups (Table 2).

### 3.3. Differences in Social-Emotional Health by History of Atopic Dermatitis Diagnosis

Children’s resilience, overall happiness, and body esteem were similar in both groups. In the AD group, the subjective health status was 80.3% for those who thought it was healthy and 3.9% for those who thought it was unhealthy, compared to 89.2% and 2.3% in the non-AD group, respectively; a high percentage of children with AD were considered unhealthy (F = 3.94, *p* = 0.026). There was a significant difference in friendship (F = 2.95, *p* = 0.007) between the AD and non-AD groups, and, although not statistically significant, 38.7% of the children with AD experienced bullying, which was slightly higher than the healthy children (30.4%) (Table 3).

## 4. Discussion

Based on the 12th data of the Korean Children’s Panel, this study was conducted to confirm the differences in the health behavior and social-emotional health status of children of late school age according to their experience of AD. In this study, 1412 households with 11-year-old children were examined, and the estimates were confirmed via weight application with samples extracted through stratified multistage extraction. Among all the subjects in this study, 8.2% were diagnosed with AD, which is lower than the 16.62% reported in a previous study [1]. Although the reason for this should be thoroughly investigated, it is thought to be due to the difference in the response to AD. Only children with active symptoms may have responded to the diagnosis experience of AD in this study, and this difference should be considered when interpreting the results.

First, we examined the differences in the general characteristics between the AD and non-AD groups. The diagnosis rate of AD in parents was significantly high, which is consistent with the common knowledge that it is a family disease. The cause of AD has not been clearly identified, but among the many assumed factors, whether the parents have a diagnosis of AD is the most obvious predictor [22]. Therefore, if parents have AD, the risk of their children having the same diagnosis is high, and it is necessary to know this and approach it proactively. In addition, environmental factors may also increase or decrease the incidence of AD [22]. Therefore, AD should be prevented by actively changing modifiable factors, such as indoor and outdoor environmental aspects and family and individual lifestyles.

Breastfeeding is known to help prevent allergic diseases, including AD, and lactation types for the first 4–6 months before the start of complementary food are especially important for child health outcomes [23]. In this study, there was no difference in infant feeding type between the AD and non-AD groups, but the transition from exclusive breastfeeding to mixed feeding was late in the former. Although some evidence suggests that breastfeeding has preventive effects on AD, a higher incidence of the disease among breastfed children has often been reported [22,24]. Scholars have suggested that some components of breast milk act as allergens according to the mother’s diet [23]. However, the reverse causal relationship error should not be overlooked. It has been found that mothers who highly recognize the advantages of breastfeeding practice it more actively [25], and it has also been found that parents with allergic diseases prefer breastfeeding and continue to practice it for a long time [26]; accordingly, higher rates of breastfeeding in groups with a high risk of AD can be misinterpreted as indicating that breastfeeding causes allergic diseases. Although there is no clear correlation between breastfeeding and AD, the overall benefits of the former are clear [23,27]. In addition, in cases of a family history of AD, the basis for the preventive effect of breastfeeding on AD has been confirmed, so it is necessary to encourage mothers to breastfeed their children if they are part of a high-risk group [23,28].

In this study, the children in the AD group had a higher intake frequency of the recommended food groups than the children in the non-AD group. This is in contrast to a previous study in which children with AD were reported to have poor nutrition due to food restrictions [5]. The excessive dietary restrictions observed in the past have recently significantly decreased after the promotion of and education on AD, and the importance of environmental management has been highly evaluated compared to dietary restrictions [29]. Similarly, in this study, it was observed that there were no restrictions on dairy products or protein-rich foods, including eggs, which are commonly restricted. In addition, the mothers in the AD group perceived their children as unhealthy, thereby maintaining positive eating habits. Although food allergies are major factors exacerbating AD in children, the causal relationship should be confirmed before restriction, and it should not be carried out indiscriminately [4]. Therefore, it is important to continuously promote and spread knowledge about AD to prevent inappropriate food restrictions and to maintain healthy eating habits.

Unlike the above report that children have healthy eating habits, the frequencies of the consumption of soda and instant food, directly reported by the children, were higher in the AD group than in the non-AD group, although the difference was not statistically significant. Food intake, such as soda, was found to increase when not managed by parents, and it was found to be influenced by peers [30]; as children’s autonomy increases with age, soda and instant food consumption could be high in school or outside in the absence of parental care. Most children with chronic renal disease regarded their own diseases as insignificant and only showed a passive response when under parental care [31]. As it is important to manage lifestyle and environment in the control of AD, it is necessary to educate and support children in recognizing their health problems and in maintaining healthy eating habits on their own. 

In this study, there was no difference in physical activity and sleep according to the diagnosis of AD. The effect of AD on physical activity is also inconsistent among previous studies. One study suggested that exercise can worsen the symptoms of AD, which may limit children’s physical activities [7]. In contrast, one study reported that children exercised more to improve their health [8]. Although the effects of AD on the physical activities of children have not been clearly identified, it has been confirmed that physical activity helps reduce children’s stress [6]. Therefore, children suffering from the disease should be encouraged to engage in enough physical activity while also aiming to reduce their exposure to worsening symptoms caused by it. Children with AD are known to have a lower sleep quality, even though their sleep times are similar to the sleep times of those without symptoms [32]. Unlike previous studies, the results of this study showed no significant difference in terms of sleep. The first possible reason for this is that the original survey only examined the time of sleep and not the quality of sleep, and the second possible reason is that the severity of AD is not reflected in the results. Therefore, further research is needed to assess the sleep behavior of children, considering the quality of sleep and the severity of AD symptoms. 

There was no difference in children’s resilience, overall happiness, or body esteem between the AD and non-AD groups. Most children with chronic diseases have been found to show a negative self-concept compared with healthy children [33]. In particular, the external skin lesions of AD have been found to make children perceive themselves as different from others and have also been found to cause alienation, as they are misunderstood as being contagious or bad [3,11]. These factors are known to negatively affect the body and self-concept. However, in this study, there was no difference in resilience, overall happiness, or body esteem between the AD and non-AD groups according to the diagnosis of AD. Positive parenting based on affection, rational explanation, and social support has an important influence on the formation of children’s self-concept [34,35]. Therefore, although AD is a risk factor in the formation of a positive self-concept, it is supposed that, in this study, a healthy self-concept was formed as a result of the parents’ positive parenting style and good family relationships acting as protective factors.

This study found that the children in the AD group had difficulties in their relationships with friends during school life. There were no other difficulties in relationships with teachers or in academic achievement. Although not statistically significant, it could be seen that the frequency of bullying experiences in the children in the AD group was higher than that in the non-AD group, which was also considered in the same context. The skin lesions of AD are the cause of bullying, and children suffering from the disease experience difficulties in their relationships with friends [3,11]. In a previous study [31,36], children with a chronic illness felt discomfort in being with friends due to dietary and activity restrictions that required their friends’ consideration, which led to distancing. In the case of children with AD, health behaviors, including diet and physical activities, are managed by their parents, which may increase their difficulties in interacting with friends. School life and peer relationships are important factors for children. In this study, the children’s self-concept was found to be positive, but, in a previous study, despite bullying and difficulties in friendship, young children under the age of 10 generally had positive self-concepts that became negative as they entered adolescence [11]. The participants of this study were 11 years old, of late school age, and they were about to enter adolescence. If the difficulties in their relationships with friends continue, it could negatively affect their self-concepts and school adjustment. Therefore, it is necessary to pay attention to the changes in children’s self-concepts and help them to maintain a positive self-concept by solving difficulties in friend interactions. Specifically, by increasing their self-care skills related to managing AD, children with AD can increase their self-efficacy and lower their negative perceptions of AD. Parents should be conscious of not only their children’s health behaviors but also their difficulties with social relationships, especially those with peers. In addition, teachers and peers should recognize that AD is not a contagious disease and that it requires dietary and activity restrictions; such an understanding can help children with AD feel a better sense of belonging in schools.

As this study used secondary data from the PSKC, there were limitations to the research methodology. First, in the 12th survey of the PSKC, since the severity and specific characteristics of AD were not confirmed, it was difficult to identify their effects in detail. Second, the health behavior variables of the children, which were only measured based on their mothers’ reports, may differ from the children’s actual behavior patterns. Third, this study analyzed children’s health behavior and social-emotional health status cross-sectionally, so it is not possible to confirm the trends of health behavior and social-emotional health status according to the diagnosis of AD. Therefore, it is necessary to identify longitudinal changes in children’s health behaviors and social-emotional health status in consideration of the onset, duration, and severity of AD in the future.

Despite the aforementioned limitations, this study is meaningful in that it investigated the differences in health behavior and social-emotional health according to the history of atopy dermatitis based on representative data, and it confirmed that there were difficulties in peer relationships despite healthy lifestyles and positive self-concepts.

## 5. Conclusions

In conclusion, in this study, 8.2% of children had a history of AD (with a high prevalence of AD among their parents), a long breastfeeding duration (in the mixed feeding group), and consumed a lot of healthy food. In addition, there were no problems with academic achievement or self-concepts, but difficulties in relationships with friends were found.

Unlike previous studies, this study showed that children with AD have similar or even better lifestyles than children without the disease. This result suggests that we should treat these children with higher standards by providing them and their parents with high-quality information and that we should target specific education programs on AD to improve their quality of life and help them better manage their symptoms. 

In terms of social-emotional health status, the mothers reported that their children were unhealthy, but the children with AD did not think negatively about themselves, showing similar levels of resilience, overall happiness, and body esteem compared to the other children. A mother’s perception of her child motivates her to care for them, and as a result, they have a healthy lifestyle and develop a positive self-concept. However, the participants of this study demonstrated difficulties with peer relationships at school. Therefore, when applying interventions, we should not solely focus on teaching children and their parents to manage the symptoms of AD but should also address the possible peer relationship problems that they may face in school.

## Figures and Tables

**Table 1:** Comparison of general characteristics in atopic dermatitis group and non-atopic dermatitis group (*n* = 1412, *N* = 469,015).

Variables	Categories	AD (*n* = 109, *N* = 38,431)	Non-AD (*n* = 1303, *N* = 430,584)	t or F * (*p*)
		*n* (%) or M ± SE	*n* (%) or M ± SE	
**Children**				
Sex	Male	61 (56.6%)	664 (51.1%)	0.57 (0.457)
	Female	48 (43.4%)	639 (48.9%)	
Height (cm)		147.97 ± 0.60	148.48 ± 0.19	0.80 (0.433)
Weight (kg)		42.21 ± 0.93	43.52 ± 0.30	1.35 (0.187)
BMI	Underweight	6 (5.0%)	76 (6.2%)	1.80 (0.179)
	Normal	81 (78.4%)	881 (69.7%)	
	Overweight/obesity	21 (16.6%)	303 (24.1%)	
Infant feeding type	Exclusive breastfeeding	50 (49.5%)	540 (47.5%)	0.16 (0.910)
	Mixed feeding	41 (39.6%)	475 (39.7%)	
	Formula milk	10 (10.9%)	161 (12.7%)	
Start of mixed feeding ^†^	1–4 month	30 (64.1%)	423 (82.5%)	5.71 (0.024)
	5–6 month	15 (35.9%)	91 (17.5%)	
**Parents**				
Employment status	Both parents	45 (43.3%)	622 (50.2%)	1.22 (0.307)
	Only father	45 (54.9%)	443 (45.6%)	
	Only mother	0 (0%)	28 (2.3%)	
	None	2 (1.8%)	14 (1.9%)	
Atopic dermatitis diagnosis	Yes	21 (19.6%)	103 (9.2%)	6.97 (0.014)
	No	79 (80.4%)	1086 (90.8%)	

* Rao-Scott test, ^†^ responses of subjects whose initial lactation type was mixed feeding; AD, atopic dermatitis group; Non-AD, non-atopic dermatitis group; n, unweighted sample size; N, weighted sample size; %, weighted %.

**Table 2:** Differences in health behavior by history of atopic dermatitis diagnosis (*n* = 1412, *N* = 469,015).

Variables	Categories	AD (*n* = 109, *N* = 38,431)	Non-AD (*n* = 1303, *N* = 430,584)	t or F * (*p*)
		*n* (%) or M ± SE	*n* (%) or M ± SE	
Dairy intake	Agree/neutral	72 (67.2%)	833 (69.8%)	0.22
	Disagree	28 (32.8%)	375 (30.2%)	(0.640)
Protein intake	Agree/neutral	96 (97.0%)	1105 (91.6%)	5.41
	Disagree	4 (3.0%)	103 (8.4%)	(0.028)
Vegetable intake	Agree/neutral	88 (88.1%)	916 (76.3%)	6.089
	Disagree	12 (11.9%)	292 (23.7%)	(0.020)
Fruit intake	Agree/neutral	91 (86.8%)	1019 (84.3%)	0.15
	Disagree	9 (13.2%)	1889 (15.7%)	(0.699)
Oil intake	Agree/neutral	96 (95.5%)	1096 (90.2%)	3.50
	Disagree	4 (4.5%)	1120 (9.8%)	(0.072)
Fat intake	Agree/neutral	88 (86.6%)	1020 (84.4%)	0.20
	Disagree	12 (13.4%)	188 (15.6%)	(0.663)
Salt intake	Agree/neutral	91 (37.2%)	1067 (33.7%)	0.19
	Disagree	9 (62.8%)	141 (66.3%)	(0.665)
Snack intake	Agree/neutral	91 (92.0%)	1067 (87.6%)	1.96
	Disagree	9 (8%)	141 (12.4%)	(0.173)
Soda intake times/week	None	20 (20.1%)	353 (27.3%)	2.57 (0.091)
	1–2/week	62 (60.6%)	629 (50.1%)	
	More than 2/week	27 (19.3%)	302 (22.5%)	
Instant food intake times/week	None	5 (6.6%)	117 (8.7%)	0.99 (0.377)
	1–2/week	61 (53.0%)	749 (59.4%)	
	More than 2/week	43 (40.4%)	418 (31.9%)	
Indoor activity days/week	Not at all	20 (16.0%)	285 (21.1%)	0.77 (0.454)
	1–3/week	51 (49.3%)	625 (49.5%)	
	More than 4/week	38 (34.7%)	393 (29.4%)	
Outdoor activity days/week	Not at all	19 (23.2%)	270 (21.3%)	0.18 (0.811)
	1–3/week	66 (57.8%)	753 (57.7%)	
	More than 4/week	24 (19.0%)	280 (21.1%)	
Sedentary time (hr)		3.26 ± 0.09	3.25 ± 0.03	−0.14
				(0.893)
Sleep time for week (hr)		9.05 ± 0.08	8.94 ± 0.02	−1.08
				(0.286)
Sleep time for weekend (hr)		9.52 ± 0.10	9.45 ± 0.03	−0.68
				(0.505)

* Rao-Scott test; AD, atopic dermatitis group; Non-AD, non-atopic dermatitis group; n, unweighted sample size; N, weighted sample size; %, weighted %.

**Table 3:** Differences in social-emotional health by history of atopic dermatitis diagnosis (*n* = 1412, *N* = 469,015).

Variables	Categories	AD (*n* = 109, *N* = 38,431)	Non-AD (*n* = 1303, *N* = 430,584)	t or F * (*p*)
		*n* (%) or M ± SE	*n* (%) or M ± SE	
Resilience		3.00 ± 0.03	2.99 ± 0.02	−0.01 (0.925)
Overall happiness		3.23 ± 0.05	3.25 ± 0.01	0.35 (0.727)
Body esteem		3.13 ± 0.06	3.06 ± 0.02	−1.36 (0.186)
Subjective health status	Healthy	88 (80.3%)	1170 (89.2%)	3.94 (0.026)
	Usual	17 (15.8%)	108 (8.5%)	
	Unhealthy	4 (3.9%)	25 (2.3%)	
Relationships with friends		3.17 ± 0.03	3.25 ± 0.02	2.95 (0.007)
Relationships with teachers		3.16 ± 0.05	3.17 ± 0.02	0.09 (0.926)
Bullying experience	Yes	41 (38.7%)	392 (30.4%)	2.86 (0.102)
	No	68 (61.3%)	892 (69.6%)	
Academic performance		4.22 ± 0.16	4.21 ± 0.06	−0.09 (0.928)

* Rao-Scott test; AD, atopic dermatitis group; Non-AD, non-atopic dermatitis group; n, unweighted sample size; N, weighted sample size; %, weighted %.

## Data Availability

Not applicable.
